# Population Attributable Fraction of Tobacco Use and Type 2 Diabetes Mellitus: An Analysis of the ENSANUT 2021

**DOI:** 10.3390/epidemiologia6040084

**Published:** 2025-12-02

**Authors:** Julio Cesar Campuzano, Jorge Martin Rodríguez, Luz Myriam Reynales, Anaid Hernández, Diana Carolina Urrego

**Affiliations:** 1 Investigador en Ciencias Médicas, Instituto Nacional de Salud Pública de México, Cuernavaca 62100, MOR, Mexico; jcampuzano@insp.mx; 2 Instituto de Salud Pública, Pontificia Universidad Javeriana, Bogotá 110231, Colombia; jrodriguez.h@javeriana.edu.co; 3 Escuela de Medicina, Universidad Latino Americana, Cuernavaca 62290, MOR, Mexico; niyita@hotmail.com; 4 Investigadora Organización Panamericana de la Salud-OPS, Bogotá 57-601, Colombia; urdiana@javeriana.edu.co

**Keywords:** tobacco use reduction, type 2 diabetes, population attributable risk, population surveys, Mexico, nested case–control studies

## Abstract

Background: Robust evidence demonstrates that tobacco use acts as a causal and, therefore, modifiable risk factor for the development of type 2 diabetes mellitus (T2DM). However, its specific population-level impact in Mexico has not yet been quantified. Objective: This study aimed to estimate the population attributable fraction (PAF) of T2DM associated with tobacco use among Mexican adults, utilizing data from the 2021 National Health and Nutrition Survey (ENSANUT). Methods: A nested case–control analysis was conducted within the complex sampling design of the ENSANUT. Adults aged 20 years or older were included. Cases were defined as individuals with a self-reported medical diagnosed T2DM diagnosis; controls were individuals without T2DM. Exposure status was categorized as current person who smokes, former person who smokes, and never person who smokes. A logistic regression model was employed, adjusting for key covariates including age, sex, socioeconomic status, and comorbidities. The PAF was subsequently calculated using the Miettinen formula. Results: The adjusted PAF for T2DM attributable to smoking was 10.1% (95% CI: 4.07–14.97). This finding suggests that approximately one in eight T2DM cases could be prevented through the elimination of tobacco use. The association was more pronounced among men and individuals with a history of heavy tobacco use. Conclusion: The estimated PAF for T2DM due to tobacco use underscores the significant contribution of policies established within the WHO Framework Convention on Tobacco Control to the prevention of chronic diseases. The implementation and strengthening of such policies, including increased tobacco taxes, comprehensive smoking bans in public places, on-package warnings, and advertising prohibitions, would prove highly beneficial. These findings show a strong population-level association between tobacco use and T2DM, but causality cannot be established. Future longitudinal studies in Mexico are needed to confirm these results.

## 1. Introduction

Mexico, a middle-income country, is experiencing an epidemiological transition characterized by increasing age-specific mortality rates in adults, attributable to chronic non-communicable diseases (NCDs) such as type 2 diabetes (T2DM) and chronic kidney disease (CKD). In 2019, NCDs accounted for 74.9% of DALYs (Disability Adjusted Life Years). Specifically for diabetes, the findings show that the burden (measured in DALYs) was higher in women than in men in 1990, but this relationship reversed over time, with the burden of diabetes being higher in men in 2019. The prevalence of diabetes has steadily increased in both men and women [1]. According to the Global Burden of Disease, in 2021, in Mexico, there were 2 million years of life lost due to premature death (YLL) related to diabetes and 1.1 million years of life lost due to disability (YLD), which resulted in 3.1 million DALYs [2].

In this context, type 2 diabetes mellitus (T2DM) represents one of the primary public health challenges worldwide. In 2021, more than 537 million adults were estimated to be living with diabetes. This number is projected to increase to 783 million by 2045 [3], which would be an increase of more than 30%.

In Mexico, the situation is particularly alarming: the national prevalence of diabetes in adults was 18.3% in 2022, with a significant proportion of undiagnosed cases [4,5]. This disease is the second leading cause of death in the country and one of the main sources of disability [6,7,8]. Tobacco use, for its part, is a well-established risk factor for multiple chronic diseases, including cardiovascular disease, cancer, and chronic obstructive pulmonary disease [9,10].

A systematic review on the worldwide prevalence of tobacco use in patients with T2DM reported, however, that some studies showed a lower probability of tobacco use in patients with T2DM compared to those without the condition. Tobacco products increase the risk of T2DM, and tobacco use increases the risk of macrovascular complications from this disease. Increased mortality and an increased risk of premature microvascular complications such as diabetic peripheral neuropathy have also been observed [11].

In addition to the above, other studies have documented an association [12,13,14,15,16]. The Health Consequences of Smoking—50 Years of Progress (2014) report explicitly establishes the causal relationship between tobacco consumption and the development of T2DM, concluding that tobacco consumption not only increases the risk of developing T2DM, but also complicates its treatment, and increases the likelihood of developing complications such as nephropathy, blindness, and amputations [13,14,15,17]. Likewise, Roderick et al. (2019) discuss previous studies reporting relative risks between 1.37 and 1.44 for the development of T2DM in person who smokes compared to non-person who smokes, where they describe plausible pathophysiological mechanisms, such as altered glucose metabolism and nicotine-induced insulin resistance [11].

Other epidemiological studies have shown that person who smokes have a 30% to 40% higher risk of developing T2DM compared to those who have never smoked [18,19,20,21]. This relationship appears to follow a dose–response pattern, and it is stronger in heavy person who smokes [22,23,24]. Additionally, another disease burden study estimated that for 2019, tobacco use has a PAF of 9.9% for T2DM [25].

At the pathophysiological level, nicotine and other compounds in tobacco smoke affect the function of pancreatic β cells, induce insulin resistance, and promote chronic inflammation, which contributes to the development of metabolic disorders [26]. In addition, smoking complicates diabetes treatment and increases the risk of micro and macrovascular complications [21].

The effects of tobacco smoke differ depending on whether exposure is active or passive. Active person who consumes tobaccos inhale smoke directly through the end of the cigarette, while passive person who consumes tobaccos inhale what is called environmental tobacco smoke (ETS), which includes a mixture of side stream smoke—which comes from the burning tip of the cigarette—and part of the exhaled mainstream smoke. Although the concentrations of ETS components are considerably lower than those of the mainstream smoke inhaled by the active tobacco person who consumes tobacco, both absorb components of tobacco smoke through the respiratory tract and alveoli [27]. As a result, passive people who consume tobacco are at greater risk of cardiovascular disease, cancer, respiratory infections, and even developing nicotine dependence [28].

In Mexico, the prevalence of tobacco use among adults aged 15 years and older was 15.6% (14.6 million adults). 24.3% of men (10.8 million) and 7.4% of women (3.5 million) used tobacco in 2023 [29].

Estimating the PAF allows us to quantify the proportion of cases of a disease that could be avoided if a specific risk factor were eliminated, under the assumption of causality supported by scientific literature. This metric is especially useful for prioritizing public policies and allocating health resources. Although there are international studies that have estimated the PAF of smoking for T2DM [11,30,31]. An equivalent analysis has not been conducted with data representative of the Mexican population.

Therefore, the present study aims to estimate the PAF of T2DM associated with smoking in Mexican adults, using data from the National Health and Nutrition Survey (ENSANUT) 2021 [32]. This estimate will help to measure the impact of tobacco use on the burden of diabetes in Mexico and to support integrated strategies for the prevention of chronic non-communicable diseases.

## 2. Materials and Methods

### 2.1. Study Design and Data Source

A nested case–control analysis was conducted within the National Health and Nutrition Survey 2021 (ENSANUT Continua 2021). This survey is representative at the national, regional, urban-rural, and federal entity levels, and uses a probabilistic, stratified, and cluster design. The ENSANUT 2021 survey collected information from approximately 12,600 households, using a sample designed to estimate key indicators of health, nutrition, and chronic diseases in the Mexican population [32].

### 2.2. Study Population

Adults aged 20 years or older with complete information on type 2 diabetes mellitus (T2DM) diagnosis and tobacco use were included. Observations with missing data on key variables or with chronological inconsistencies (e.g., smoking onset age later than current age) were excluded.

### 2.3. Dependent Variable: Diabetes Diagnosis

The outcome variable was the presence of T2DM, defined by [self-reported medical diagnosis (“Has any doctor told you that you have diabetes or high blood sugar?”). A dichotomous variable was generated that takes the value of 1 for people diagnosed with T2DM and 0 for those who did not report this condition. The subsample with blood glucose measurement in the ENSANUT 2021 study corresponds to approximately 33% of the adults surveyed, that is, nearly 4200 people. This subsample was specifically designed to estimate the prevalence of undiagnosed diabetes and assess glycemic control in individuals with a previous diagnosis. However, it has important limitations for its use as a diagnostic exclusion condition, small sample size, nature of the diagnosis, and an objective different from the original design. To strengthen the validity of the results, in addition to the self-reported diagnosis of type 2 diabetes mellitus (T2DM), a sensitivity analysis was performed using the biochemical criteria available in ENSANUT 2021: blood glucose and glycated hemoglobin (HbA1c).

For these reasons, we believe that using self-reported medical diagnosis from the general questionnaire offers a more suitable basis for identifying potential cases and controls. This approach allows for complete national representativeness, the inclusion of individuals with a confirmed diagnosis, a larger sample size, and the possibility of adjusting for multiple covariates available in the survey.

### 2.4. Primary Exposure Variable: Active Tobacco Use

The main exposure was tobacco use, constructed from the variables (current use) and (history of use). Three mutually exclusive categories were defined:

Current person who smokes: people who reported smoking every day or some days (1 or 2). Former person who smokes: people who do not currently smoke [3] but reported having smoked in the past (1 or 2). Never person who smokes: people who do not currently smoke and have never smoked [3]. These categories were coded into a dichotomous variable (tobacco) with values 1 for current and former person who smokes, 0 for non-person who smokes. This decision was based on pathophysiological evidence demonstrating that the risk of developing T2DM is related to cumulative exposure to tobacco, rather than current consumption status. The effects of smoking on glucose metabolism, insulin sensitivity, and pancreatic β-cell function have been documented to persist even after smoking cessation, justifying considering former person who smokes as an exposed population. Moreover, this coding improved the stability of the multivariate model and yielded more significant estimates of the association (more robust ORs), suggesting that the impact of smoking as a risk factor is manifested by exposure time rather than current status.

### 2.5. Classification by Intensity of Tobacco Use

To assess the dose–response gradient, a consumption intensity variable was constructed based on the average number of cigarettes smoked per day. The variable used was cigarettes per day, and if it was empty, it was imputed from the variable cigarettes per week.

This variable allowed independent models to be created for each intensity category, with the aim of estimating the population attributable fraction (PAF) specific to the intensity of exposure. All people who smoke were considered in this classification.

### 2.6. Covariates

Sociodemographic variables and clinical history were included as potential confounders: age, sex, socioeconomic status, residence (urban/rural), geographic region, excessive alcohol consumption, obesity, cardiovascular disease, high blood pressure, kidney disease, hypertriglyceridemia, and hypercholesterolemia. All comorbidities were measured by self-reported medical diagnosis, based on direct questions in the individual adult health questionnaire (ENSANUT 2021). Participants responded to questions about whether a doctor had previously diagnosed them with these conditions, which allowed for the identification of chronic diseases based on the medical diagnosis reported by the respondent. This approach is consistent with the survey design and has been validated in previous studies as a useful tool for estimating disease burden in large, representative populations.

### 2.7. Statistical Analysis

A logistic regression model adjusting for complex sampling design (svyset) was used to estimate both crude and adjusted odds ratios (ORs) for the association between tobacco use and T2DM. Separate models were run to compare tobacco use categories (current person who smokes, former person who smokes, never person who smokes) and smoking intensity categories (low, moderate, high). We used stata version 15.0 to analysis the data.

### 2.8. Estimation of the Population Attributable Fraction (PAF)

The PAF was estimated using the Miettinen formula, suitable for case–control studies: FAP = Pe(OR − 1OR)FAP = Pe\left(\frac{OR − 1}{OR}\right) [33].

Where PeP_e is the proportion of individuals exposed among cases (current person who smokes or former person who smokes), and OR is the adjusted Odds Ratio obtained from the logistic model. The 95% confidence intervals for PAF were estimated using the delta method, which has been widely applied in epidemiological studies for attributable risk calculations. This formula assumes a causal relationship between exposure and outcome, and is valid under the condition of rarity of the event [34]. Global and stratified PAFs were calculated by smoking intensity, with the aim of evaluating the dose–response attributable risk as tobacco use intensity increases.

### 2.9. Ethical Considerations

This study is classified as risk-free, considering that secondary, aggregated, and anonymous databases were used without direct contact with individuals or the collection of primary information that would allow their identification. ENSANUT 2021 is a public, anonymized, and freely accessible database. No additional informed consent was required. However, it is necessary to highlight that the various aspects and ethical considerations of ENSANUT, information collection procedures, and its instruments, were evaluated and approved by the Research Ethics Committee of the National Institute of Public Health of Mexico (Ensanut 2021 CI: 1750 code: 2230 folio: S1-21) [32].

## 3. Results

Data from 13,302 adult Mexicans included in the ENSANUT 2021 study were analyzed. A weighted prevalence of T2DM of 12.63% was found, and the proportion of current and former person who smokes among cases was 38.0%, which allowed estimating the population attributable fraction (PAF) based on the adjusted models (Table 1).

### 3.1. Association Between Smoking and Diabetes

In the model adjusted for the total population, smoking was significantly associated with an increased risk of T2DM (OR = 1.36, 95% CI: 1.12–1.65, *p* = 0.002). This result indicates that person who smokes are 36% more likely to report a diagnosis of diabetes compared to those who have never smoked, adjusted for age, sex, area of residence, region, and alcohol consumption. In the present study, people with other comorbidities were not excluded.

Table 2 shows the covariates that adjusted the model; all were statistically significant. For example, for the survey population, the risk of diabetes increased by 6% for every increase in risk. Similarly, the risk of diabetes in women was 48% higher than in men; those living in urban areas had almost 1.3 times the risk of T2DM compared to those living in rural areas. These results were statistically significant (*p* < 0.05).

Nevertheless, when the smoking status was analyzed separately, current smokers showed a non-significant association with T2DM (ORa = 1.22; 95% CI: 0.91–1.63; *p* = 0.181), while former smokers had a significantly higher risk compared to never smokers (ORa = 1.47; 95% CI: 1.18–1.83; *p* = 0.001).

### 3.2. Analysis by Intensity of Use

When stratified by intensity of tobacco use, a dose–response pattern was observed in the risk of T2DM: Low consumption (<10 cig/day): OR = 1.39 (95% CI: 1.14–1.68, *p* = 0.001); Moderate consumption (10–19 cig/day): OR = 1.44 (95% CI: 1.16–1.79, *p* = 0.001); High consumption (≥20 cig/day): OR = 1.44 (95% CI: 1.16–1.80, *p* = 0.001). These results show a dose–response relationship, where the risk of diabetes increases with the intensity of tobacco use, although the differences between levels were not statistically significant.

**Figure 1:**
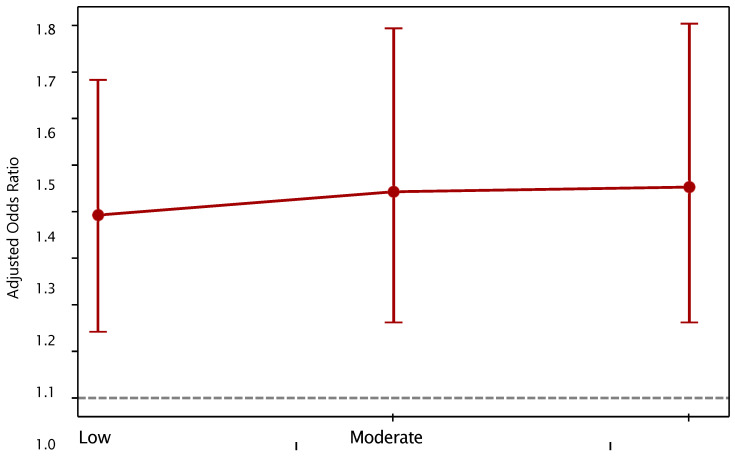
Dose–Response Curve of Tobacco Use. Note: Each model was adjusted for age, sex, region, area, and alcohol consumption. The increasing pattern of OR reinforces a dose–response relationship.

Finally, the risk of T2DM increased slightly by intensity of consumption: from 1.39 when the intensity was low to 1.44 when it was moderate or high (Figure 1).

### 3.3. Estimation of the Population Attributable Fraction (PAF)

Using Miettinen’s formula: PAF = Pe(OR − 1OR)PAF = P_e\left(\frac{OR − 1}{OR} \right); where PeP_e is the proportion of exposed individuals among cases, and aOR is the adjusted odds ratio (Table 3), the following PAF were estimated:

PAF (%)  PAF = Pe × (OR − 1)/OR

The highest PAF was observed in the high consumption group, suggesting, under the assumption of causality, that up to 1 in 8 cases of type 2 diabetes in this subgroup could be attributed to tobacco use. In all models, the 95% confidence intervals for the OR of smoking excluded unity, and the *p*-values were statistically significant at <0.01, which supports the robust association. The consistency of the results in the stratified models reinforces the validity of the findings.

A sensitivity analysis using the ENSANUT 2021 biomarkers (glucose and glycosylated hemoglobin HbA1c), showing that the prevalence of DM2 based on self-reports was 11.0%; by incorporating biomarkers, an additional 4.6% of undiagnosed cases were identified, raising the total prevalence to 15.6%; in this alternative scenario, the association between smoking and DM2 remained robust, with an adjusted OR of 1.34 (95% CI: 1.10–1.61); and the population attributable fraction (FAP) estimated with biochemical criteria was 9.8% (95% CI: 3.9–14.4), very close to that obtained with self-reported diagnosis (10.1%). These results confirm that the findings are consistent regardless of the definition of the outcome, reinforcing the strength of the main conclusions.

## 4. Discussion

The results show that smoking is significantly associated with an increased risk of T2DM, and that this association persists even after adjusting for various social and demographic factors. The estimated PAF suggests that between 10% and 12.5% of T2DM cases in Mexico could be attributed to tobacco use, representing a significant and potentially preventable burden.

This study estimated for the first time the population attributable fraction (PAF) of type 2 diabetes mellitus (T2DM) attributable to smoking in Mexican adults, using representative data from the ENSANUT 2021. These findings are consistent with the international literature, especially with research that estimated a PAF of 9.9% [25]. Other studies show how tobacco use can increase the risk of T2DM by up to 30% in diverse populations [19,23,35,36,37]. Furthermore, a dose–response relationship has been observed, as identified in this analysis, where the attributable risk increases with the intensity of cigarette consumption [21,26]. This consistency strengthens the validity of the results.

Other studies show that the PAF of smoking for T2DM varies among different populations (but also around 10%), reflecting variations in consumption patterns, risk profiles, and prevalence of other risk factors (overweight, obesity, physical inactivity, alcoholism, among others). Even in the second study, they report how the PAF of tobacco use, which, although potentially lower compared to obesity or physical inactivity, can generate interaction with these risk factors, generating greater morbidities for people [38,39].

From a methodological perspective, the use of a nested case–control analytical approach within a population-based survey with complex sampling, such as the ENSANUT, represents a robust and efficient strategy for estimating associations and calculating the PAF. This robust design allows valid inferences to be made about the adult Mexican population (≥20 years), making it an ideal source for epidemiological studies. This analysis takes advantage of the complex sample structure of ENSANUT (2021) to identify cases (individuals with a self-reported medical diagnosis of T2DM) and controls (without a diagnosis), maintaining population representativeness and reducing potential selection bias. This approach is particularly useful in large-scale surveys, as it allows associations between exposures (such as smoking) and outcomes (T2DM) to be estimated without the need for a separate study, optimizing resources and time.

Furthermore, the nested design facilitates adjustment for multiple covariates available in the ENSANUT (age, sex, socioeconomic status, comorbidities), which improves the internal validity of the analysis. The wealth of sociodemographic and clinical information contained in the ENSANUT allows for exploring interactions and performing stratified analyses, such as those presented in our study.

Although the cross-sectional design limits potential causal inference, measures were taken to enhance temporal validity, such as comparing the age at which smoking began and the age at which diabetes was diagnosed. Additionally, the Miettinen formula used to calculate the PAF is appropriate for this type of analytical approach, and has been widely validated in other epidemiological studies [34]. In our case, it is not only methodologically feasible but also adds value to the analysis by allowing for accurate population estimates of the impact of smoking on the burden of T2DM in Mexico.

Estimating Population Attributable Fractions (PAFs) has direct implications for public health planning. In Mexico, where T2DM prevalence is up to 18.3% [4] and tobacco use affects over 19% of adults [40]; a PAF of 10% suggests hundreds of thousands of cases could be prevented. Effective prevention interventions, such as increasing the tobacco tax, could be key [41,42]. Furthermore, evidence shows that fiscal policies on tobacco have a greater impact on people with noncommunicable diseases (NCDs) like diabetes, reinforcing the need to integrate tobacco prevention strategies into metabolic disease control programs [43,44].

It is important to note that the PAF estimation using Miettinen’s formula assumes that the outcome is relatively rare. Given the prevalence of T2DM in Mexico (12–18%), this assumption may not strictly hold, which could slightly affect the precision of our estimates. However, previous studies have shown that the formula remains valid for outcomes with moderate prevalence, and our results are consistent with those reported in other populations.

The study’s strengths include its use of a national database, detailed classification of tobacco use by intensity, and adjustment for various covariates. According to data from ENSANUT (2021), the prevalence of T2DM in Mexican adults was 11.0% by self-reported medical diagnosis, that is, people who reported having been diagnosed by a health professional. However, when biochemical measurements such as fasting glucose and glycated hemoglobin (HbA1c) were incorporated, an additional 4.6% prevalence of undiagnosed diabetes was identified, raising the total estimated prevalence to 15.6%.

This difference reveals that one in three cases of diabetes in Mexico is undiagnosed, underscoring the importance of complementing [self-reported medical diagnosis with biomarkers to obtain a more accurate estimate of the prevalence and distribution of the disease. These findings are consistent with observations from other Latin American countries; in Brazil, a study based on national survey data estimated that tobacco use accounted for approximately 13.5% of the PAF for cardiovascular disease [45]. In Colombia, tobacco use, abdominal obesity, and physical inactivity have been linked as significant risk factors for T2DM [46,47]. Studies from Argentina, Brazil, Chile, and Mexico also report a substantial tobacco-attributable burden, particularly in cardiometabolic disease diseases [48].

Limitations include the cross-sectional nature of the design, potential information bias generated by self-reporting, and the lack of information on passive tobacco smoke exposure. However, the consistency of the results and the magnitude of the estimated PAF support the relevance of these findings; also, the finding of a consistent and significant association between consumption and intensity with the risk of diabetes reinforces the biological plausibility and relevance of considering tobacco as a priority metabolic risk factor. We acknowledge that relying on self-reported diagnosis may underestimate T2DM prevalence, since biochemical data suggest a substantial number of undiagnosed cases. Additionally, due to the cross-sectional design, temporality cannot be assured and causality cannot be inferred.

It can be stated that smoking represents a significant fraction of the burden of T2DM in Mexico. Estimating the PAF based on national data provides a key tool for public health decision-making, including the evaluation of fiscal policies, cessation campaigns, and attributable cost models. This study reinforces the need to address smoking not only as a cardiovascular risk factor, but also as a high-priority metabolic determinant.

This work therefore offers a quantitative tool for health decision-making, from including the tobacco component in interventions for the prevention of chronic non-communicable diseases to the economic justification for stricter fiscal measures against tobacco. This analysis demonstrates that moving toward a smoke-free population could translate not only into fewer heart attacks and cancer, but also into less diabetes.

In a country where the burden of diabetes continues to rise, and where tobacco use affects millions of adults, recognizing and quantifying this intersection is a health imperative. The present PAF estimate can be converted, thusly, into a decisive step toward a more integrated, preventative, and equitable policy framework for public health in Mexico. They also underscore the importance of public health interventions aimed at tobacco control as a comprehensive strategy that includes reducing the burden of disease caused by T2DM. The results of this study should be interpreted within the context of global efforts to reduce the burden of NCDs, particularly those associated with tobacco use, as well as T2DM, cardiovascular disease, cancer, and chronic respiratory diseases.

At the international level, the WHO Framework Convention on Tobacco Control (FCTC) is the first global public health treaty, adopted in 2003 and in force since 2005, which establishes evidence-based measures to reduce both the supply of and demand for tobacco products. This legal instrument has been ratified by more than 180 countries and reaffirms the right of all people to the highest attainable standard of health. In addition, WHO has developed the MPOWER technical package, which includes six key measures for implementing the FCTC at the national level: monitoring tobacco use, protecting the population from smoke, providing help to quit smoking, warning about the dangers, enforcing advertising bans, and increasing tobacco taxes. These actions have proven to be cost-effective and have contributed to reducing smoking prevalence in various contexts.

In parallel, the WHO has promoted the Global Commitment to NCDs 2020–2030, which seeks to accelerate the prevention and control of these diseases through multisectoral interventions, strengthening health systems, and promoting healthy public policies. This framework recognizes that tobacco control is a cornerstone of achieving Sustainable Development Goal (SDG) target 3.4, which aims to reduce premature mortality from NCDs by one-third by 2030.

In conclusion, based on this evidence, we consider that the use of the population attributable fraction (PAF) in our study is relevant to estimate the population impact of smoking on the burden of T2DM in Mexico. While there is robust evidence of causal association, the cross-sectional nature of the study limits direct causal inference in our sample.

The findings of our study reinforce the need to integrate tobacco control policies into national NCD prevention strategies. The estimation of the population attributable fraction (PAF) of T2DM associated with smoking in Mexican adults demonstrates the potential impact of these policies on reducing the disease burden. Furthermore, it underscores the importance of strengthening epidemiological surveillance, tobacco market regulation, and cessation campaigns as part of a comprehensive response.

## Figures and Tables

**Table 1:** Sociodemographic characteristics of the cases and their controls from the ENSANUT 2021.

Variables	Control			Cases			Total		
	*n* *	%	[CI 95%]		%	[CI 95%]		%	[CI 95%]
History of tobacco									
Non-smoker	7823	63.6	[62.3, 64.9]	1091	62.7	[59.1, 66.2]	8914	63.5	[62.2, 64.7]
Current smoker	2001	19.7	[18.6–20.8]	183	14.3	[11.6–17.6]	2184	19.1	[18.1, 20.2]
Former smoker	1887	16.8	[15.8–17.8]	325	23.0	[19.7–26.6]	2212	17.4	[16.5, 18.4]
Sex									
Man	4730	48.6	[47.3, 49.9]	517	41.6	[38.5, 44.9]	5247	47.9	[46.7, 49.0]
Woman	7062	51.4	[50.1, 52.7]	1093	58.4	[55.1, 61.5]	8155	52.1	[51.0, 53.3]
Area									
Rural	2823	20.1	[18.8, 21.6]	363	17.7	[15.4, 20.3]	3186	19.9	[18.5, 21.3]
Urban	8969	79.9	[78.4, 81.2]	1247	82.3	[79.7, 84.6]	10,216	80.1	[78.7, 81.5]
Region									
North Pacific	1466	9.5	[8.6, 10.6]	189	8.9	[6.9, 11.4]	1655	9.5	[8.5, 10.5]
Frontier	855	12.9	[11.7, 14.3]	125	13.3	[10.5, 16.8]	980	13.0	[11.8, 14.3]
Pacific-Central	967	11.0	[10.4, 11.7]	118	9.4	[7.4, 11.8]	1085	10.9	[10.3, 11.4]
North Central	2595	12.6	[12.0, 13.2]	372	12.3	[10.7, 14.2]	2967	12.6	[12.1, 13.1]
Center	909	9.7	[9.1, 10.5]	124	11.8	[8.6, 15.9]	1033	10.0	[9.1, 10.9]
Cd. Mexico	1226	8.2	[7.8, 8.7]	177	8.8	[7.3, 10.6]	1403	8.3	[7.9, 8.7]
Edo. Mexico	1101	13.7	[13.0, 14.5]	153	13.0	[10.9, 15.5]	1254	13.7	[13.0, 14.3]
South Pacific	1180	12.2	[11.1, 13.4]	164	13.9	[11.7, 16.4]	1344	12.4	[11.3, 13.5]
Peninsula	1493	10.0	[9.4, 10.6]	188	8.6	[7.1, 10.5]	1681	9.9	[9.3, 10.4]
Alcohol consumption									
Daily	58	0.4	[0.3, 0.6]	9	0.5	[0.2, 1.1]	67	0.4	[0.3, 0.6]
Weekly	904	9.0	[8.3, 9.9]	45	2.7	[1.8, 4.0]	949	8.4	[7.7, 9.2]
Monthly	1096	10.9	[10.0, 11.8]	74	6.0	[4.2, 8.4]	1170	10.3	[9.5, 11.2]
No consume	9734	79.7	[78.6, 80.7]	1482	90.8	[88.3, 92.8]	11,216	80.9	[79.8, 81.8]
Total	11,792	100.0		1610	100.0		13,402	100.0	

* n: total number.

**Table 2:** Association between smoking status and type 2 diabetes mellitus in Mexican adults. Adjusted model, ENSANUT 2021 (*n* = 13,302).

Variable	ORa	CI 95%	*p*
Current smokers (vs. never)	1.22	0.91–1.63	0.181
Former smokers (vs. never)	1.47	1.18–1.83	0.001
Age (years)	1.06	1.05–1.06	<0.001
Women (Ref: Men)	1.48	1.26–1.74	<0.001
Urban residence	1.27	1.04–1.54	0.018
Alcohol consumption (weekly)	0.36	0.14–0.93	0.034

Note: Adjusted odds ratios (ORa) estimated using multivariate logistic regression. Reference category for smoking variables: never smokers. Model adjusted for age, sex, region, area (urban/rural), and alcohol consumption. Standard errors and confidence intervals adjusted for ENSANUT’s complex survey design.

**Table 3:** Population attributable fraction (PAF) of type 2 diabetes mellitus related to tobacco use, according to intensity of consumption, ENSANUT 2021.

Exposure Subgroup	ORa	IC 95% [ORa]		PeP_e	PAF (%)	CI 95% PAF	
Total population	1.36	1.12	1.65	0.38	10.10%	4.07	14.97
Low consumption	1.39	1.14	1.68	0.32	9.00%	3.93	12.95
Moderate consumption	1.44	1.16	1.79	0.35	10.70%	4.83	15.45
High consumption	1.44	1.16	1.80	0.41	12.50%	5.66	18.22

Note: DPF calculated with Miettinen’s formula. ORs obtained from fitted models. Values that can be interpreted as a proportion of cases attributable to smoking under causal assumptions.

## Data Availability

The data sets analyzed or generated during this study are publicly available and were obtained from the National Survey of Health and Nutrition (ENSANUT) Continuous 2021. The information was downloaded directly from the official website of the National Institute of Public Health (INSP) at the following URLs: This ensures full transparency and reproducibility of the research findings, as all primary data sources are clearly referenced.

## Abbreviations

The following abbreviations are used in this manuscript:

CI	Confidence Intervals
CKD	Chronic kidney disease
DALYs	Disability Adjusted Life Years
ENSANUT	National Health and Nutrition Survey from México
ETS	Environmental tobacco smoke
NCDs	Chronic non-communicable diseases
OR	Odds Ratios
ORa	Adjusted Odds Ratios
PAF	Population Attributable Fraction
PeP_e	Proportion of Individuals Exposed Among Cases
T2DM	Type 2 diabetes mellitus
WHO	Word Health Organization
YLD	Years of life lost due to disability
YLL	Years of life lost due to premature death

## References

[1] Guerrero-López C.M., Serván-Mori E., Miranda J.J., Jan S., Orozco-Núñez E., Downey L., Feeny E., Heredia-Pi I., Flamand L., Nigenda G. (2023). Burden of non-communicable diseases and behavioural risk factors in Mexico: Trends and gender observational analysis. J. Glob. Health.

[2] Ong K.L., Stafford L.K., McLaughlin S.A., Boyko E.J., Vollset S.E., Smith A.E., Dalton B.E., Duprey J., Cruz J.A., Hagins H. (2025). Global, regional, and national burden of diabetes from 1990 to 2021, with projections of prevalence to 2050: A systematic analysis for the Global Burden of Disease Study 2021. Lancet.

[3] International Diabetes Federation (2023). IDF Diabetes Atlas (10th ed.). https://diabetesatlas.org.

[4] Basto-Abreu A., López-Olmedo N., Rojas-Martínez R., Aguilar-Salinas C.A., Moreno-Banda G.L., Carnalla M., Rivera J.A., Romero-Martinez M., Barquera S., Barrientos-Gutiérrez T. (2023). Prevalencia de prediabetes y diabetes en México: Ensanut 2022. Salud Publica De México.

[5] Basto-Abreu A.C., López-Olmedo N., Rojas-Martínez R., Aguilar-Salinas C.A., De la Cruz-Góngora V.V., Rivera-Dommarco J., Shamah-Levy T., Romero-Martínez M., Barquera S., Villalpando S. (2021). Prevalence of diabetes and glycemic control in Mexico: National results from 2018 and 2020. Salud Pública De México.

[6] Agudelo-Botero M., Dávila-Cervantes C.A. (2025). Mortality and Years of Life Lost from Cardiometabolic Diseases in Mexico: National and State-Level Trends, 1998–2022. Public Health Rep..

[7] Rojas-Martínez R., Escamilla-Nuñez C., Aguilar-Salinas C., Castro-Porras L., Romero-Martínez M., Lazcano-Ponce E. (2024). Trends in the mortality of diabetes in Mexico from 1998 to 2022: A joinpoint regression and age-period-cohort effect analysis. Public Health.

[8] Bello-Chavolla O.Y., Antonio-Villa N.E., Fermín-Martínez C.A., Fernández-Chirino L., Vargas-Vázquez A., Ramírez-García D., Basile-Alvarez M.R., Hoyos-Lázaro A.E., Carrillo-Larco R.M., Wexler D.J. (2022). Diabetes-Related Excess Mortality in Mexico: A Comparative Analysis of National Death Registries Between 2017–2019 and 2020. Diabetes Care.

[9] Noubiap J.J., Nansseu J.R., Endomba F.T., Ngouo A., Nkeck J.R., Nyaga U.F., Kaze A.D., Bigna J.J. (2019). Active smoking among people with diabetes mellitus or hypertension in Africa: A systematic review and meta-analysis. Sci. Rep..

[10] Reynales-Shigematsu L.M. (2016). Tobacco and cancer: Epidemiology and new perspectives of prevention and monitoring in Mexico. Salud Pública De México.

[11] Roderick P., Turner V., Readshaw A., Dogar O., Siddiqi K. (2025). The global prevalence of tobacco use in type 2 diabetes mellitus patients: A systematic review and meta-analysis. Diabetes Res. Clin. Pract..

[12] Huh Y., Han K., Choi M.J., Kim J.H., Kim S.M., Nam G.E. (2022). Association of smoking status with the risk of type 2 diabetes among young adults: A nationwide cohort study in South Korea. Nicotine Tob. Res..

[13] Akter S., Goto A., Mizoue T. (2017). Smoking and the risk of type 2 diabetes in Japan: A systematic review and meta-analysis. J. Epidemiol..

[14] Chang S.A. (2012). Smoking and type 2 diabetes mellitus. Diabetes Metab. J..

[15] Campagna D., Alamo A., Di Pino A., Russo C., Calogero A.E., Purrello F., Polosa R. (2019). Smoking and diabetes: Dangerous liaisons and confusing relationships. Diabetol. Metab. Syndr..

[16] Chi Y., Wang X., Jia J., Huang T. (2022). Smoking status and type 2 diabetes, and cardiovascular disease: A comprehensive analysis of shared genetic etiology and causal relationship. Front. Endocrinol..

[17] U.S. Department of Health and Human Services (2014). The Health Consequences of Smoking—50 Years of Progress: A Report of the Surgeon General.

[18] Dastani M., Ghorbani M., Eskandarioun M., Hassani Goodarzi T., Torabian A., Talebnia S.E. (2025). Association Rule Mining Analysis of Cardiovascular Risk Factors in the CDC Diabetes Health Indicators Dataset. Infosci. Trends.

[19] Willi C., Bodenmann P., Ghali W.A., Faris P.D., Cornuz J. (2007). Active smoking and the risk of type 2 diabetes: A systematic review and meta-analysis. JAMA.

[20] Loretan C.G., Cornelius M.E., Jamal A., Cheng Y.J., Homa D.M. (2022). Cigarette smoking among US adults with selected chronic diseases associated with smoking, 2010–2019. Prev. Chronic Dis..

[21] CDC Centers for Disease Control and Prevention Archive (2023). Tobacco Control Interventions. Health.

[22] Flor L.S., Anderson J.A., Ahmad N., Aravkin A., Carr S., Dai X., Gil G.F., Hay S.I., Malloy M.J., McLaughlin S.A. (2024). Health effects associated with exposure to secondhand smoke: A Burden of Proof study. Nat. Med..

[23] Pan A., Wang Y., Talaei M., Hu F.B., Wu T. (2015). Relation of active, passive, and quitting smoking with incident type 2 diabetes: A systematic review and meta-analysis. Lancet Diabetes Endocrinol..

[24] Lycett D., Nichols L., Ryan R., Farley A., Roalfe A., Mohammed M.A., Szatkowski L., Coleman T., Morris R., Farmer A. (2015). The association between smoking cessation and glycaemic control in patients with type 2 diabetes: A THIN database cohort study. Lancet Diabetes Endocrinol..

[25] Bai J., Shi F., Ma Y., Yang D., Yu C., Cao J. (2022). The global burden of type 2 diabetes attributable to tobacco: A secondary analysis from the global burden of disease study 2019. Front. Endocrinol..

[26] World Health Organization (2023). Tobacco and Diabetes Internet. https://iris.who.int/bitstream/handle/10665/375763/9789240088580-spa.pdf?sequence=1&isAllowed=y.

[27] Samet J.M. (2002). Los riesgos del tabaquismo activo y pasivo. Salud Pública De México.

[28] Agudo L.S. (2004). El fumador pasivo. Monogr. Tab..

[29] Encuesta Global de Tabaquismo en Adultos (GATS) (2023). México 2023. Gobierno de México, Departamento de Prevención y Control del Tabaquismo. https://portal.insp.mx/control-tabaco/reportes/encuesta-global-de-tabaquismo-en-adultos-gats-mexico-2023.

[30] Wang M., Maimaitiming M., Zhao Y., Jin Y., Zheng Z.J. (2024). Global trends in deaths and disability-adjusted life years of diabetes attributable to second-hand smoke and the association with smoke-free policies. Public Health.

[31] Guo D., Yu Y., Zhu Z. (2025). Global burden of type 2 diabetes attributable to secondhand smoke: A comprehensive analysis from the GBD 2021 study. Front. Endocrinol..

[32] Romero-Martínez M., Barrientos-Gutiérrez T., Cuevas-Nasu L., Bautista-Arredondo S., Colchero M.A., Gaona-Pineda E.B., Martínez-Barnetche J., Alpuche-Aranda C., Gómez-Acosta L.M., Mendoza-Alvarado L.R. (2021). Metodología de la Encuesta nacional de Salud y nutrición 2021. Salud Pública De México.

[33] Llorca J., Fariñas-Álvarez C., Delgado-Rodríguez M. (2001). Fracción atribuible poblacional: Cálculo e interpretación. Gac. Sanit..

[34] Greenland S., Drescher K. (1993). Maximum likelihood estimation of the attributable fraction from logistic models. Biometrics.

[35] Yang Y., Peng N., Chen G., Wan Q., Yan L., Wang G., Qin Y., Luo Z., Tang X., Huo Y. (2022). Interaction between smoking and diabetes in relation to subsequent risk of cardiovascular events. Cardiovasc. Diabetol..

[36] Liu G., Hu Y., Zong G., Pan A., E Manson J., Rexrode K.M., Rimm E.B., Hu F.B., Sun Q. (2020). Smoking cessation and weight change in relation to cardiovascular disease incidence and mortality in people with type 2 diabetes: A population-based cohort study. Lancet Diabetes Endocrinol..

[37] Durlach V., Vergès B., Al-Salameh A., Bahougne T., Benzerouk F., Berlin I., Clair C., Mansourati J., Rouland A., Thomas D. (2022). Smoking and diabetes interplay: A comprehensive review and joint statement. Diabetes Metab..

[38] Marbaniang S.P., Lhungdim H., Chauhan S., Srivastava S. (2021). Interaction of multiple risk factors and population attributable fraction for type 2 diabetes and hypertension among adults aged 15–49 years in Northeast India. Diabetes Metab. Syndr. Clin. Res. Rev..

[39] Rajaobelina K., Dow C., Mancini F.R., Dartois L., Boutron-Ruault M., Balkau B., Bonnet F., Fagherazzi G. (2019). Population attributable fractions of the main type 2 diabetes mellitus risk factors in women: Findings from the French E3N cohort. J. Diabetes.

[40] Ramírez-García D., Fermín-Martínez C.A., Sánchez-Castro P., Núñez-Luna A., Basile-Alvarez M.R., Fernández-Chirino L., Vargas-Vázquez A., Díaz-Sánchez J.P., Kammar-García A., Almeda-Valdés P. (2024). Smoking, all-cause, and cause-specific mortality in individuals with diabetes in Mexico: An analysis of the Mexico city prospective study. BMC Public Health.

[41] Chaloupka F.J., Yurekli A., Fong G.T. (2012). Tobacco taxes as a tobacco control strategy. Tob. Control.

[42] Scollo M., Younie S., Wakefield M., Freeman J., Icasiano F. (2003). Impact of tobacco tax reforms on tobacco prices and tobacco use in Australia. Tob. Control.

[43] Reynales-Shigematsu L.M., Sáenz-de-Miera B., Llorente B., Maldonado N., Shanon G., Jha P. (2022). Benefits of the cigarette tax in Mexico, by sex and income quintileBenefícios do imposto sobre cigarros no México: Análise por sexo e quintil de renda. Rev. Panam. Salud Publica.

[44] Milián R.P., Enríquez M.E.A. (2021). Programas de intervención aplicados en población mexicana para la prevención de enfermedades no transmisibles 2010–2020. Anu. Investig. UM.

[45] Ferrante D., Virgolini M. (2007). Encuesta Nacional de Factores de Riesgo 2005: Resultados principales: Prevalencia de factores de riesgo de enfermedades cardiovasculares en la Argentina. Rev. Argent. Cardiol..

[46] Acosta Ruiz L.X., Merchán M.A., Orjuela Vargas L. (2023). Diabetes mellitus tipo 2: Latinoamérica y Colombia, análisis del último quinquenio. Rev. Med..

[47] Rodríguez M., Mendoza M.D. (2019). Factores de riesgo de diabetes mellitus tipo 2 en población adulta. Barranquilla, Colombia. Rev. Colomb. Endocrinol. Diabetes Metab..

[48] Acosta L.D., Molinatti F., Peláez E. (2019). Comparison of mortality attributable to tobacco in selected Latin American countries. Población Salud Mesoamérica.

